# Immune response and insulin signalling alter mosquito feeding behaviour to enhance malaria transmission potential

**DOI:** 10.1038/srep11947

**Published:** 2015-07-08

**Authors:** Lauren J. Cator, Jose E. Pietri, Courtney C. Murdock, Johanna R. Ohm, Edwin E. Lewis, Andrew F. Read, Shirley Luckhart, Matthew B. Thomas

**Affiliations:** 1 Grand Challenges in Ecosystem and Environment, Department of Life Sciences, Imperial College London, Silwood Park; 2 Department of Medical Microbiology and Immunology, University of California, Davis; 3 Department of Infectious Disease in College of Veterinary Medicine and Odum School of Ecology, University of Georgia; 4 Center for Infectious Disease Dynamics, Pennsylvania State University; 5 Department of Entomology, University of California, Davis.

## Abstract

Malaria parasites alter mosquito feeding behaviour in a way that enhances parasite transmission. This is widely considered a prime example of manipulation of host behaviour to increase onward transmission, but transient immune challenge in the absence of parasites can induce the same behavioural phenotype. Here, we show that alterations in feeding behaviour depend on the timing and dose of immune challenge relative to blood ingestion and that these changes are functionally linked to changes in insulin signalling in the mosquito gut. These results suggest that altered phenotypes derive from insulin signalling-dependent host resource allocation among immunity, blood feeding, and reproduction in a manner that is not specific to malaria parasite infection. We measured large increases in mosquito survival and subsequent transmission potential when feeding patterns are altered. Leveraging these changes in physiology, behaviour and life history could promote effective and sustainable control of female mosquitoes responsible for transmission.

Transmission of malaria parasites is inextricably linked to vector survival and feeding behaviour. Mosquitoes infected with malaria parasites have been shown to exhibit changes in feeding persistence, duration, and probing behaviour^1^. Intriguingly, changes in host-seeking behaviour are differentially associated with stages of parasite development, with decreased host-seeking associated with pre-infectious oocyst stages of the parasite^2^ and increased response with the infectious sporozoite stage^2^,^3^. Such behavioural changes are predicted to increase transmission success^1^,^4^ and have long been considered a classic example of parasitic manipulation in which malaria parasites actively alter the behaviour of the mosquito to increase their own fitness^5^,^6^. However, we recently reported that challenge with heat-killed *Escherichia coli* triggered similar time-specific changes in host-seeking propensity, suggesting that altered host-seeking behaviour is a response to immune challenge rather than a specific consequence of malaria parasite infection^2^.

Despite the potential importance of these behavioural phenomena in transmission, the underlying mechanisms remain unclear. Previous work has demonstrated that changes in the probing rates of some vectors could be attributed to parasite induced lesions in the salivary glands^7^, and one study reported altered protein expression in the heads of sporozoite infected females^8^. However, a mechanistic connection between the presence of malaria parasites in the midgut and stage-specific changes in mosquito feeding motivation and host-seeking rates is lacking. Elucidating this mechanism could help to both clarify the role of the parasite in driving the behavioural response and reveal potential novel targets for genetic manipulation of mosquito host-seeking behaviour.

Here, we sought to further investigate the role of the mosquito immune response in altered host-seeking behavior and to reveal potential mechanisms linking immune response to host-seeking propensity. Using heat-killed *E. coli* as a non-pathogenic immune elicitor, we found that the behavioural phenotype requires a challenge at the time of bloodmeal and also that it dynamically responds to dose. When we investigated the mechanism of this dynamic relationship between bloodmeal and immune challenge, we found that both heat-killed *E. coli* and the human malaria parasite, *P. falciparum,* trigger changes in insulin signaling in the mosquito midgut which are functionally linked to feeding propensity. Finally, we quantified the potential impact of altered feeding patterns on mosquito survival and hence, transmission potential.

## Results and Discussion

We began by dissecting the relationship between immune challenge and bloodfeeding. As in previous work, we challenged female *Anopheles stephensi* by injecting heat-killed *E. coli* immediately following the first bloodmeal^2^. This technique has been shown to generate both the behavioural and neurophysiological phenotypes associated with malaria parasite infection^2^, and represents a more tractable experimental approach for evaluating mosquito behaviour in the lab. This technique was especially appropriate for our aim of investigating the role of the mosquito immune response to challenge in the absence of potentially confounding pathogen dynamics^9^.

**Figure 1:**
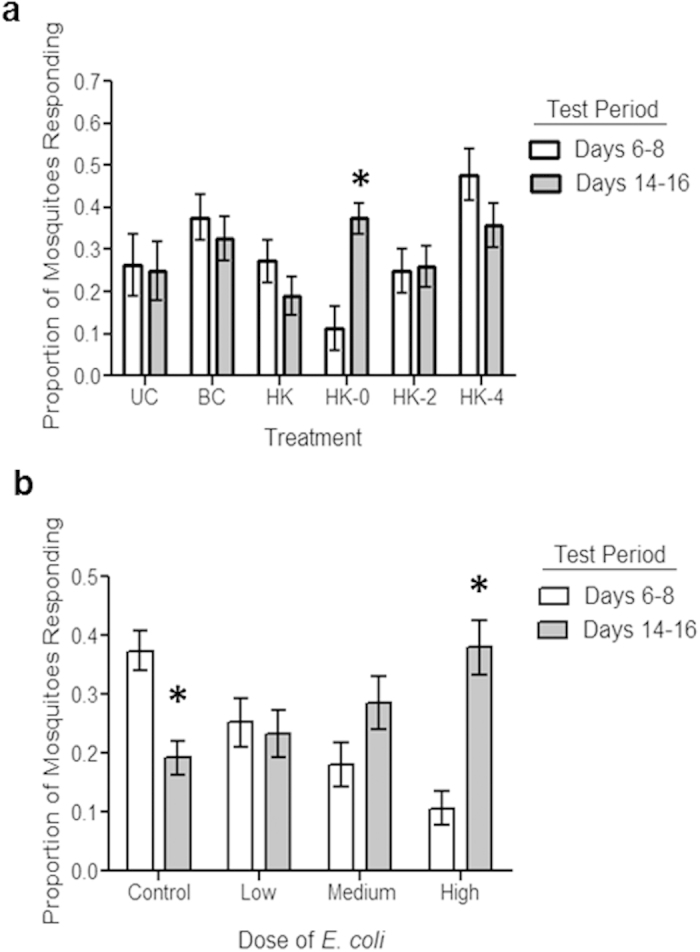
Effects of timing and dose of immune challenge on host-seeking behaviour. **A**. Immune challenge coinciding with the bloodmeal generates the mosquito host-seeking patterns associated with malaria parasite infection. The previously described ‘manipulation’ phenotype is only recapitulated when females are challenged immediately following the bloodmeal (HK-0, n = 163). Immune challenge alone (HK, n = 156) or occurring later (HK-2 days, n = 145 and HK-4 days, n = 148) do not generate the same phenotype. Controls included unmanipulated females (UC, n = 78) and females which only received a bloodmeal (BC, n = 157) This experiment was replicated twice. **B.** The effect of dose of heat-killed *E. coli* on behaviour. Dose had no effect on the duration or time of decreased response period (Fig. S2). Control, n = 391, Low, n = 227, Medium, n = 209, High, n = 221. This experiment was replicated three times. Error bars represent 1 SE and * indicates P < 0.05 between first and second test period within a treatment.

First, we examined possible interactions between the timing of immune challenge and the bloodmeal. Female mosquitoes were given an uninfected bloodmeal and were then challenged with 200,000 heat-killed *E. coli* administered either immediately following the bloodmeal, 2 days, or 4 days post-bloodmeal^2^. The behavioural response of these mosquitoes to human host cues was compared with mosquitoes that received either a bloodmeal or immune challenge alone, or were completely unmanipulated. Behaviour was assayed at two time points coinciding with non-infectious (6–8 days post infection) and infectious (14–16 days post infection) stages of parasite development in malaria-infected mosquitoes. Behavioural changes only occurred when the bloodmeal and immune challenge were synchronous (treatment x test period, Wald χ^2^ = 11.753, d.f. = 5, P = 0.04, Fig. 1a). Neither immune challenge alone nor a bloodmeal alone altered host-seeking propensity compared to unmanipulated controls, and only females challenged directly after the bloodmeal exhibited a significantly different phenotype from bloodfed control females (Fig. 1a, Wald χ^2^ = 10.91, d.f. = 11, P < 0.01).

To further investigate the role of the mosquito immune response, we examined whether the magnitude of immune response altered host-seeking patterns. We challenged bloodfed females directly after an uninfected bloodmeal with low, medium, and high doses of heat-killed *E. coli.* These doses were chosen based on previous work demonstrating that challenge with 200,000 Heat-killed *E. coli* (the “high” treatment here) generated a behavioural and neurophysiological response equivalent to that elicited by challenge with the rodent malaria parasite, *P. yoelii*^2^. We confirmed that these doses triggered corresponding variation in immune response by measuring total body expression of the immune gene *defensin* (*DEF1*) from 6–48 h post-challenge. We observed a dose effect on immune gene expression at the peak of *DEF1* expression, 12 hr post-immune challenge (Fig. S1, Table S1). Corresponding analysis of behavioural responses of females (total of 1453 independent observations) revealed that the dose of immune challenge received at the time of the bloodmeal was an important determinant of host-seeking patterns (dose x stage, χ^2^ = 20.041, d.f. = 11 , P < 0.001, Fig. 1b). The higher the dose of heat-killed *E. coli* a treatment group received, the lower its response to the host 6–8 days post-challenge. When mosquitoes from the same treatment groups were assayed on days 14–16 post bloodmeal we observed the opposite trend, with the response of mosquitoes to host odour increasing with the dose of heat-killed *E. coli*. Thus, behavioural change depended on both the dose and timing of immune challenge relative to the bloodmeal, suggesting possible trade-offs or constraints between immune response, digestion, and potentially reproduction.

With this new insight, we next set out to characterize the regulation of the altered host-seeking behaviour. Insulin-like peptides (*ILP*s) have been reported to regulate the sensitivity of odorant receptor neurons (ORNs) in *Drosophila*^10^. We previously reported that changes in the sensitivity of the ORNs in mosquito maxillary palps are associated with rodent malaria parasite infection and challenge with heat-killed *E. coli*^2^. Further, the expression levels of *ILP3* and *ILP4* in the *An. stephensi* midgut and head are altered in response to the human malaria parasite, *P. falciparum* in the ingested bloodmeal^11^. Therefore, we hypothesized that changes in *ILP* expression might be associated with observed changes in host-seeking behaviour.

**Figure 2:**
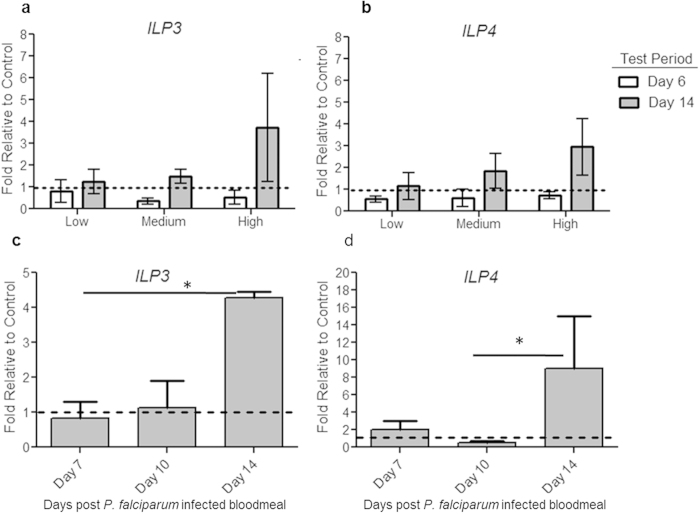
Immune challenge alters time-specific *ILP* expression in the mosquito midgut. **A-B.** The expression of *ILP* 3 (**A**) and *ILP* 4 (**B**) in the midgut in response to the high, medium, and low doses of heat-killed *E.coli* over 14 days. Horizontal dashed lines represent control levels of expression. **C-D.** Expression of *ILP* 3 (**C**) and *ILP* 4 (**D**) in the midgut is downregulated in *P. falciparum-*infected *An. stephensi* during the period of oocyst development and upregulated during the period of sporozoite development. Mosquitoes were dissected 7, 10, or 14 days after infection with *P. falciparum* and expression of *ILP* 3 or *ILP* 4 was analyzed by qRT-PCR. Both experiments were replicated independently 3 times with pools of 25 female mosquitoes. * indicates significant difference in mean expression between test periods relative to control levels (ANOVA with Tukey’s multiple comparisons) and all error bars represent 1 SE.

When we measured expression of *ILP3* and *ILP4* in the midgut and head of mosquitoes challenged with the same three doses of heat-killed *E. coli*, we found that *ILP3* (F_1,12_ = 7.56, P = 0.02) and *ILP4* (F_1,12_4.67, P = 0.05) expression levels were low in the midgut at days 6–8 post bloodmeal when host-seeking was reduced and elevated at 14–16 days (Fig. 2a,b) when host-seeking was enhanced^2^. In contrast to the midgut, *ILP3* expression in the head was elevated at day 6 relative to day 14, (d.f = 1, F = 6.35, P = 0.03; Fig. S3), but there was no significant pattern of *ILP4* expression in the head in response to any *E. coli* challenge. These results suggest that ILP expression in the midgut, not the head, regulates behavioural changes.

To confirm whether insulin signalling also plays a role in the *Plasmodium* parasite*-*triggered behavioural changes, we measured expression levels of *ILP3* and *ILP4* in the midguts of *P. falciparum*-infected *An. stephensi* at times consistent with altered host-seeking behaviour. We used the NF54 strain of *P. falciparum,* which regularly produces low-intensity infection similar to those observed in field populations^12^ (39–67% prevalence and intensity of 1.36–2.00 oocyst/midgut, Table S2). We found a sequential under- and overexpression of these *ILP*s coinciding with the sequential changes in host-seeking behaviour (Fig. 2). ILP3 expression was lowest at 7 days post-infection, but began to rise by 10 days, and was significantly elevated at 14 days post-infection (Fig. 2c; ANOVA, d.f = 2, F = 6.31, P = 0.03; Tukey’s multiple comparison test, day 7 vs day 14, P < 0.05). *ILP4* expression was lowest at day 10 post-infection and significantly increased on day 14 post-infection (Fig. 2d; ANOVA, d.f = 2, F = 5.18, P = 0.05; day 10 vs day 14, P < 0.05). Therefore, these rather diverse challenges (bacteria/transient, parasite/infective) both trigger similar changes in the expression of these two *ILP*s in the mosquito midgut.

To determine whether the association of reduced midgut *ILP* expression with host-seeking behaviour was functionally significant, peptide levels of target ILPs were knocked down by treating females with antisense *ILP3* and *ILP4* morpholino oligomers or with a control morpholino^11^. This minimally invasive method allows for targeted, efficient knockdown of midgut expression. We confirmed knockdown efficiency using a Western blot (Fig. S4). These females were then provided access to an uninfected bloodmeal. Females treated with either *ILP3* or *ILP4* morpholinos were significantly less likely to engorge on available blood (43.2% feeding and 56.3% feeding, respectively) than those treated with a control morpholino (67.1% feeding) (χ^2^ = 16.65 , d.f. = 2 , P < 0.05). Hence, reduced *ILP*3 and *ILP*4 expression is functionally associated with reduced feeding behaviour, a pattern consistent with the oocyst-stage phenotype in parasite-infected females and also females after a transient activation of the immune response with heat-killed *E. coli*^2^.

Transient activation of the mosquito immune response has now been shown to trigger the same behavioural, neurophysiological^2^, and midgut insulin signalling as active infection with malaria parasites. Parasites can manipulate their host through induction of host trade-offs^13^. However, we would argue that a more parsimonious interpretation is that the observed relationships between immune response and feeding behaviour are a mosquito response to physiological or resource-based constraints^14^. Without any mechanism specific to *Plasmodium* parasites, it seems most reasonable to conclude that changes in host-seeking behaviours in malaria parasite-infected females are a mosquito-mediated response to infection rather than parasite-driven manipulation. In fact, the development times of malaria parasites (extrinsic incubation period) might have been shaped via selective forces associated with altered feeding to maximize their likelihood of transmission.

**Figure 3:**
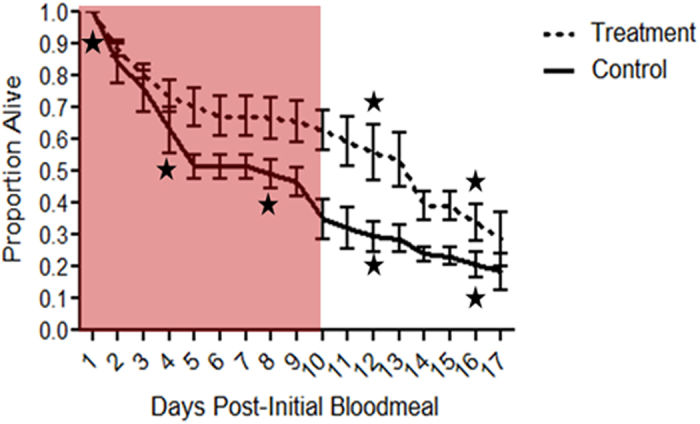
The effect of blood feeding on the cumulative proportional survival of female mosquitoes challenged with heat-killed *E. coli*. Control females received bloodmeals every 4 days while treatment females skipped the 2^nd^ and 3^rd^ bloodmeals during the period associated with down-regulated feeding behaviour. When not on bloodmeals, females were maintained on 2.5% sugar solution. Female survival was individually tracked. Each treatment group contained 100 females and the experiment was replicated twice (mean shown for total of 400 females, 200 females per treatment group). Red shading indicates the extrinsic incubation period of *P. falciparum* at 27 °C^36^. Stars below control and above treatment line indicate that treatment received a bloodmeal. Error bars represent 1 SE. Survival was compared using a Cox Proportional Hazards Test.

These conclusions do not alter the fact that the malaria parasites might benefit from these changes. Therefore, we quantified the potential impact of altered feeding behaviour for malaria transmission potential. Specifically, we examined the significance of reduced mosquito feeding and associated mortality during the early, non-infectious stages for malaria transmission potential. Bloodfeeding is known to be costly to uninfected mosquitoes^15^,^16^, but its effect on infected or immune challenged mosquitoes remains unexplored. Immune-challenged females were split into two groups, one that was bloodfed at a regular 4-day cycle and one that was not offered bloodmeals during the second and third cycles (days 4 and 8, consistent with the ‘manipulation’ phenotype). We found that skipping bloodmeals led to a significant increase in survival (Cox Regression, z = 2.37, P = 0.02), doubling the number of mosquitoes alive at the fourth and fifth feeding cycles when, in infected mosquitoes, parasites would have completed development and could potentially be transmitted (Fig. 3). Given that feeding and reproductive costs (e.g. feeding-associated mortality from defensive host behaviour and flights to shuttle between hosts and oviposition sites) are likely higher in the field than under benign laboratory conditions, these results confirm the potential importance of the altered feeding phenotype for malaria transmission. Future work quantifying the effect of altered feeding using local vector-parasite pairings under field conditions, will further clarify effect on transmission. However, this simple experiment demonstrates the need to better understand this phenomenon and its mechanisms.

We demonstrate that the interactions between immunity and behaviour appear, at least in part, to be mediated by alterations in *ILP* biology in the midgut. In *Caenorhabditis elegans* and in *D. melanogaster*, the intestine or midgut is considered an “insulin signalling center,” directing physiological responses among midgut cells and between cells of the midgut and other tissues to regulate lifespan and oxidative stress^17^. Studies in *D. melanogaster* suggest that *ILP*s produced in neurosecretory cells act as endocrine signals to control hunger, physical activity and mating^18^,^19^. In addition to regulating these important processes, *ILPs* also appear to coordinate trade-offs between these processes^20^. For example, experiments in fruit flies have revealed activation of Toll signalling in the fat body inhibits insulin signalling activity in other tissues^19^. Conversely, activation of insulin signalling in *An. stephensi* can inhibit NF-κB-dependent immunity in the midgut^21^. Previous work in mosquitoes has demonstrated that *ILP*s have been shown to play a role in the regulation of vitellogenesis and bloodmeal digestion^22^,^23^. It appears that in *An. stephensi* there is also an inhibition of insulin signalling activity in the migut in association with immune activation and additionally, that this suppression dramatically alters mosquito life history and transmission potential. We have observed that genetic manipulation of midgut insulin signalling can significantly affect both lifespan and immunity. Now we extend this phenotypic control to behaviour^24^,^25^.

Overall, our results further support the idea that the subset of mosquitoes responsible for malaria transmission exhibit altered foraging phenotypes^1^,^26^,^27^,^28^ leading to changes in mortality schedules and enhanced transmission potential^4^. Further mechanistic characterization of behavioural modifications could improve our ability to control malaria transmission in three key ways. First, incorporation of these altered behaviours into standard epidemiological models could improve our understanding and ability to predict transmission dynamics. Second, altered behavioural ecology of the subset of mosquitoes responsible for transmission provides opportunities for focusing control tools to target transmission instead of entire mosquito populations. Increased specificity of control efforts could be used to effectively control malaria transmission, while weakening the strong selection for resistance that accompanies population-wide control measures^29^. Finally, this study identifies possible new targets for genetic manipulation. Insulin signalling pathways may be used to target the parasite both directly via mosquito immunity and indirectly by altering mosquito feeding behaviour to curtail transmission potential.

## Methods

### Mosquito Rearing

All experiments were conducted using an Indian wild type strain of *An.stephensi* originally sourced from the Walter Reed Army Institute of Medical Research. *An.stephensi* used in timing, dose, and survival assays were reared at the PSU insectary at a larval density of 400 larvae per 1 L tray of distilled water. Eggs from over 1000 colony females were placed in plastic trays (25 × 25 x 7 cm) filled with 1.5 L of distilled water and held at 26 °C rearing temperature. Upon reaching 2^nd^ instar larvae were transferred to fresh trays at a density of 400 larvae per 1.5 L of distilled water. We fed larvae 10 mg of ground fish flakes (TetraFin, Melle, Germany) a day. We collected pupae and placed them in cages for emergence. Larvae for the *ILP 3/4* expression work and engorgement assays were reared at UC Davis and were provided with a 2% solution of 2:1 Sera Micron® powdered fish-fry food (Sera North America, Montgomeryville, PA) through day 4 post-hatching and then were reared on high protein, low fat Game Fish Chow pellets (Purina Mills, St. Louis, MO) until pupation. All adults were held at 26 °C , 80% RH with a 12:12 hr light:dark lighting cycle. Emerged adults were maintained on cotton pads soaked in a 10–20% sucrose solution.

### Timing of Immune Challenge

We investigated the effect of immune challenge timing relative to the bloodmeal using two control and four treatment groups (Fig. 1a). Controls included unmanipulated females that were not bloodfed or challenged (UC) and females that were not bloodfed, but challenged with heat-killed *E. coli* (HK, tetracycline resistant GFP expressing dh5 alpha strain^2^). Treated females were offered a bloodmeal on uninfected anesthetized mice (C57 BL/6, Animal Care and Use Committee of the Pennsylvania State University, permit no: 27452), then divided into the following four treatment groups: (1) no immune challenge (BC), (2) challenged with heat-killed *E. coli* immediately following bloodmeal (HK 0), (3) challenged with heat-killed *E. coli* 2 days after the bloodmeal (HK 2), and (4) challenged with heat-killed *E. coli* on day 4 post-bloodmeal (HK 4). All females receiving bacterial challenge were injected with 200,000 heat-killed *E. coli* using a microcapillary glass needle inserted intrathoracically into the anepisternal cleft^2^,^30^. Prior to challenge all groups were anesthetized on ice. Previous experiments established that injury alone did have a significant effect on this set of behaviours^2^.

The female behavioural response to a host was tested using the short range assay described in^2^. Briefly, females were aspirated individually into a release cage. This cage was connected to a cage containing the hand of a human host (LJC) with a plastic tube (12 cm diameter, 48 cm length). The 4 minute trial began when a flap blocking entry to the tube from the release cage was lifted. A positive response was counted if females exited the release cage, entered the host cage, and attempted to feed on the host. Females that did not respond within the 4 minute trial period were counted as not responding. Reponses were tested on days corresponding with oocyst (days 6–8) and sporozoite (13–15) stages of *Plasmodium yoelii* infection at this temperature. The responses of 40 individual females per treatment group were tested for each stage (80 females total per treatment group). This experiment was replicated twice with the exception of the unmanipulated control group, which was added for only the second replicate of the experiment. The Pennsylvania State University Institutional Review Board determined that the experiments whereby mosquitoes were attracted to LJC’s hand did not meet the criteria for human subjects research and thus, do not require human subjects approval.

### Dose of Immune Challenge and Response

For these studies, 3–5 day old females provided an uninfected mouse bloodmeal were divided into two control and three treatment groups. Controls included an unmanipulated group to account for any impact of cold anesthetization and an injury control group that was cold anesthetized and injected with sterile LB. The three treatment groups were all cold anesthetized and challenged with varying doses of heat-killed *E. coli* directly after the bloodmeal. The low challenge group received a dose of 2,000 heat-killed *E. coli*, the medium challenge group received 20,000 heat-killed *E. coli*, and the high challenge group received 200,000 heat-killed *E. coli*. The high dose is equivalent to the concentration used in the timing experiment (Fig. 1b) and in previous experiments using heat-killed *E. coli* challenge and has been shown to produce similar changes in behaviour to challenge with a *P. yoelii-*infected bloodmeal^2^. The responsiveness of these females to human odor was measured using the short-range assay described above (Fig. 1b). In the first replicate, responses were measured during two periods; days 6–7 and days 14–15. In the second and third replicate, we measured responses in an additional intermediate period between these and report responsiveness on days 6–8, 10–12, and 14–16 post initial bloodmeal. We tested a naïve group of 20–25 females per treatment group on each day.

### Immune Gene Expression

To quantify the response of females to different doses of heat-killed *E. coli*, we sampled 10 females 0, 6, 12, 24 and 48 hrs post immune challenge. Females were killed immediately with chloroform and stored in RNA*later* RNA stabilization reagent and held at 4 °C. Five females were sampled from each treatment group at each time point (N = 125 females) and individually prepared in *B*-Mercaptoethanol and RLT lysis buffer (Qiagen, Venlo, Netherlands) for messenger RNA isolation. mRNA was extracted and quantified for each female as described in^9^,^30^. cDNA from each experimental sample was quantified by comparing threshold cycle numbers against a standard curve generated from 1:10 serial dilutions of our standard sample (cDNA from a pool of four mosquitoes). Levels of cDNAs for both *DEF1* and *ribosomal protein S7* (*rps7*), a housekeeping gene commonly used in a wide range of studies on *Anopheles*^31^,^32^,^33^, were quantified from individual mosquitoes collected across all experimental treatments relative to a standard curve produced for that gene (Fig. S1). To account for individual differences in background gene expression, *rpS7* cDNA counts were included as a covariate in our statistical analyses (see below). Primers and probes were previously described^30^.

### ILP 3 and ILP 4 expression in female challenged with heat-killed E. coli and P. falciparum

Females from control, low, medium, and high treatment groups from the dose experiment were sampled on days 6–7 post-challenge and day 14 post-challenge with *E. coli*, or days 7, 10, and 14 post-infection with *P. falciparum* (11NF54 strain). We dissected heads and midguts from 20–30 females per treatment into PBS and tissues were stored in Trizol reagent (Invitrogen, Carlsbad, CA). Samples were homogenized by pulse sonication and total RNA was isolated using Trizol (Invitrogen) according to the manufacturer’s protocol. Quantitative RT-PCR was performed on an ABI 7300 Sequence Detection System (Applied Biosystems; Foster City, California) using *ILP*-specific primers and probes as previously described^19^. Data were analyzed using the delta-delta Ct method and *ILP* expression in challenged/infected groups was normalized against *rps7* to control for loading then normalized against levels in controls receiving blood only (Fig. 2). Experiments were replicated three times and data are reported as fold change in expression relative to controls.

### Knockdown of ILP 3 and ILP4 using Morpholinos

As described in^34^ Anti-*ILP3* (5′ TCGTGGACGACA TCTTGACAGAGGT-3′) or anti-*ILP4* (5′-CGTGGAACTTTCATCTCAAGGACCT-3′) morpholinos (Gene Tools, Philometh, OR) at a stock concentration of 500 μM were diluted to 10 μM in saline and ATP (Sigma Aldrich, St. Louis, MO) was added to a final concentration 1 mg/ml as a feeding stimulant. Immediately prior to feeding, morpholino solutions were warmed in a water bath at 37 °C for 10 min. Vivo-morpholino standard control targeting an intron in the human beta-globin gene (5′-CCTCTTACCTCAGTTACAATTTATA-3′) in saline at equal concentrations was used as a matched control. Cohorts of 50–60 newly emerged female mosquitoes were provided with water only (no sucrose) for 24 hours and then provided a meal of either control-morpholino, *ILP3*-morpholino, or *ILP4*-morpholino in saline via a Hemotek artificial circulation system (Discovery Workshops, Accrington, UK). After 30 min, any unfed mosquitoes were removed from experimental cohorts.

### Verification of ILP knockdown

To validate the efficiency of morpholino-mediated peptide knockdown *in vitro* (Fig. S4), we used an experimental surrogate of tagged *As*ILP3 and *As*ILP4 overexpression in rat pancreatic islet or insulinoma cells to infer analogous activity against peptide levels in *A. stephensi*. These cells express properly processed, mature rat insulin^35^ and we have confirmed that processed, mature *As*ILPs can also be produced by these cells following transfection (not shown). Here, rat insulinoma cells (RIN-5F, ATCC, Manassas, VA) were plated at a density of 250,000 cells/ml in RPMI 1640 growth medium (Invitrogen) supplemented with 10% FBS and 5% glucose and allowed to rest until adherent. On day 1, cells were transfected with *As*ILP3- or *As*ILP4-encoding plasmid (pcDNA3.1/V5-His, Invitrogen) using Effectene reagent (Qiagen, Limburg, Netherlands) according to the manufacturer’s protocol. The *As*ILP constructs contained twelve base pairs of the 5′ untranslated region for morpholino binding, the full-length *As*ILP coding sequence, and a C-terminal V5 epitope tag for detection. Constitutive, high level transcription was driven by the cytomegalovirus (CMV) promoter. Empty plasmid was used as a negative control. On day 2, cells were treated with either standard control, anti-*AsILP3* or anti-*AsILP4* morpholinos (10 μM, Gene Tools) by directly adding morpholinos to the culture medium. On day 3, cells were collected into cell extraction buffer (Invitrogen), mixed with sample loading buffer (125 mM Tris-HCl pH 6.8, 10% glycerol, 10% SDS, 0.006% bromophenol blue) and boiled for 5 minutes. These experiments were replicated with 2 independent preparations of cells. To validate the efficiency of morpholino-mediated peptide knockdown *in vivo* (Fig. S4), we analyzed *As*ILP3 and *As*ILP4 levels in whole mosquitoes fed control or anti-*As*ILP morpholinos by western blot, as peptide levels in individual tissues are low and difficult to quantify. Three days after feeding of morpholinos, whole mosquitoes were collected into cell extraction buffer (Invitrogen) and homogenized with a pestle by grinding for 2–3 minutes. Homogenates were then passed through a QIAshredder column (Qiagen), cleared by centrifugation at 16,000 g for 10 minutes, mixed with sample loading buffer (125 mM Tris-HCl pH 6.8, 10% glycerol, 10% SDS, 0.006% bromophenol blue) and boiled for 5 minutes. This experiment was performed using 15–20 mosquitoes from 1 morpholino feed. Proteins from *in vitro* and *in vivo* experiments were separated on 16.5% Tris-tricine gels (BioRad) and transferred to nitrocellulose membranes (BioRad). Membranes were then fixed with 0.05% glutaraldehyde for 5 minutes and blocked with 5% non-fat dry milk in TBS-T for 2 hours at room temperature. For detection of peptides, membranes were incubated overnight at 4 °C with a 1:5,000 dilution of anti-V5 antibody (Sigma Aldrich) or 1:1,000 dilution of anti*-As*ILP antibody (Antibodies Inc., Davis, CA) in 5% non-fat dry milk. Membranes were then washed three times with TBS-T for 5 minutes and incubated with a 1:10,000 dilution of secondary HRP-conjugated rabbit anti-mouse antibody (Sigma Aldrich) in 5% non-fat dry milk overnight. Proteins were visualized by incubating membranes with SuperSignal West Dura chemiluminescent reagent (Pierce, Rockford, IL) for 3 minutes and exposing on an Image Station 4000 Pro digital imager (Kodak, Rochester, NY) for 5–10 minutes. Densitometry was performed using the ImageJ gel analysis tool (http://rsbweb.nih.gov/ij/) and values were normalized to Coomassie brilliant blue stain for total protein.

### Measurement of Engorgement Success in Morpholino-treated Mosquitoes

Mosquitoes were maintained on cotton pads soaked in 10% sucrose through day 3 post- morpholino feeding, then starved on water for 24 h and subsequently provided a meal of human red blood cells in PBS (Cellgro, Manassas, VA) via Hemotek. Mosquitoes were allowed to engorge on blood for 15 min. After 15 min, mosquitoes were examined for the presence of blood in the midgut, counted and discarded. Experiments were independently replicated three times and data are reported as the proportion of mosquitoes that engorged in 15 min.

### Survival of immune challenged females

As described above, 200 females were challenged directly after a bloodmeal (HK-0) on an uninfected mouse with 200,000 heat-killed *E. coli* (high dose). Each female was individually transferred to a 50 mL falcon tube fitted with a mesh lid and placed in a 27 °C incubator. Females were continually provided 2–5 ml of water for oviposition at the bottom of each tube and a cotton ball soaked in a 2.5% sugar solution. We used this lower concentration of sugar to impose some stress on these females, which otherwise were not subjected to many common sources of stress or mortality likely experienced in the field (long range flight, predation, host defensive behaviour, environmental fluctuation). Female mortality was checked daily. Females in the control group were provided a bloodmeal from an uninfected anesthetized mouse every 4 days (days 4, 8, 12, and 16). Treatment females were offered bloodmeals on days 12 and 16, but skipped the meals associated with down-regulated feeding behaviour in our assays (day 4 and 8 feds). The study was carried out in strict accordance with the recommendations in the guide for the Care and Use of Laboratory Animals of the National Institutes of Health. The protocol was approved by the Animal Care and Use Committee of the Pennsylvania State University (#44512). To increase engorgement rate, sugar was removed the day prior to a bloodmeal and returned following the bloodmeal. Females which did not engorge were removed from analyses. We tracked females for one day beyond the final bloodmeal days (Fig. 3). The experiment was replicated twice giving a final sample size of 400.

### Data Analysis

For the timing experiment, we utilized a Generalized Linear Model (GLM) fitted with a binary logistic regression to determine the effect of treatment on the likelihood that females responded positively to the host. We determined the statistical significance of treatment (unmanipulated control/ bloodfed control/ heat-killed only/ heat-killed Day 0/ heat-killed day 2/heat-killed day 4), time period (days 6–8/ days 13–15), day, replicate, wing length and the interactions of these variables. For the dose experiment, we also utilized a GLM fitted with a binary logistic regression. We determine the effect of treatment (unmanipulated control/injured/ 2,000 *E. coli*, 20,000 *E. coli*, and 200,000 *E. coli*), test period (days 6–8, 10–12, or 14–16), day, replicate, and wing length and the interaction of these variables on the probability that females responded.

We ran a general linear mixed effects model to estimate how *DEF1* expression was influenced by dose of immune challenge, the time point the immune system was sampled post-immune challenge, and their interaction. We first transformed *DEF1* cDNA levels to ensure a normally distributed error structure. We included immune challenge treatment (unmanipulated, injured, 2,000 *E. coli*, 20,000 *E. coli*, and 200,000 *E. coli*) and sampling time point (6 hr, 12 hr, 24 hr, 48 hr) post-immune challenge as fixed effects in the model analysis. To control for inter-sample variation introduced through the sampling, mRNA extraction, and cDNA conversion processes, we normalized *DEF1* expression by including centered *rpS7* cDNA levels as a covariate in our model analysis. To control for inter-block variation, biological replicate was included in the model as a random effect. Model fit and assumption of equal variances was confirmed through model residuals.

For analysis of *ILP* expression, raw data were rank transformed and analyzed by two-way ANOVA to examine the effects of both time and dose on *ILP* expression in *E. coli*-challenged mosquitoes. Statistical analysis of *ILP* expression in *P. falciparum*-infected mosquitoes consisted of non-parametric, one way ANOVA followed by Tukey’s multiple comparison test. Assays of mosquito engorgement were replicated three times and control groups from each replicate were compared by chi-squared test to control for variation between cohorts. No significant differences between the three replicates were found (P > 0.05). Data from all replicates were then combined for chi-squared goodness of fit analysis to determine differences in the proportion of mosquitoes engorged in control and anti-*ILP* morpholino-treated groups.

Finally, the survival of immune-challenged females under different feeding regimes was compared using a Cox Regression. We tested for the effect of feeding treatment, replicate, and interaction between these variables.

In all cases, full models were reduced through stepwise elimination of non-significant interactions and terms. Significance values reported for significant terms were those taken from the final model. Significance values reported for non-significant terms were those computed in the final step prior to removal of the term from the model. Non-significant values were only reported if they had a significant interaction with another parameter.

**Data Deposition:** All data has been deposited in the Dryad Online Repository doi:10.5061/dryad.qn765.

## Additional Information

**How to cite this article**: Cator, L. J. *et al.* Immune response and insulin signalling alter mosquito feeding behaviour to enhance malaria transmission potential. *Sci. Rep.* **5**, 11947; doi: 10.1038/srep11947 (2015).

## Supplementary Material

Supplementary Information
