# Supplementary material for: Immune response and insulin signalling alter mosquito feeding behaviour to enhance malaria transmission potential
Source: Sci Rep. 2015 Jul 8;5:11947. doi: 10.1038/srep11947 (PMC4495552; doi:10.1038/srep11947)
Supplement: Supplementary Information [file srep11947-s1.pdf]

Supplemental Information

Immune challenge and insulin signalling alter mosquito feeding behaviour to enhance malaria transmission potential

Lauren J. Cator*†, Jose E. Pietri†, Courtney C. Murdock, Edwin Lewis, Andrew F. Read, Shirley Luckhart, Matthew B. Thomas

†These authors contributed equally to this work.
* Corresponding author. E-mail: l.cator@imperial.ac.uk

The effect of feeding regime on survival. There was no significant interaction between feeding treatment and replicate (Cox Regression, P>0.05). There was however a significant replicate effect between the two experiments (Fig. 1, Cox Regression, z=-3.65, P<0.01). Controlling for this significant variation, we still found that feeding treatment significantly predicted survival (Cox Regression, z=-2.37, P=0.02).

Effects of *P. falciparum* infection on *ILP* expression. Our assays of *ILP* gene expression in response to heat-killed *E. coli* demonstrated that immune challenge triggers long-term changes in midgut ILP levels that are in line with changes in host-seeking behaviour. To explore the relevance of these effects in the context of malaria parasite infection in the mosquito, we measured the expression of *ILP3* and *ILP4* in the midgut on days 7, 10, and 14 post-infection with *P. falciparum* (Fig. 2ab). We found that expression patterns were consistent with the effects observed in *E. coli* challenged mosquitoes. During the oocyst state of infection (7-10 days) *ILP* levels were decreased, while during the infectious sporozoite stage of infection (14 days), *ILP* levels were increased. Specifically, *ILP3* expression was lowest at 7 days post-infection, but began to rise by 10 days and was significantly elevated at 14 days post-infection (Tukey's multiple comparison test,7 days vs 14 days, P<0.05). *ILP4* expression was lowest at day 10 post-infection and significantly increased on day 14 post-infection (Tukey's multiple comparison test, 10 days vs 14 days, P<0.05).

The effect of anti-*ILP* morpholino treatment on engorgement success. Our previous experiments demonstrated that low *ILP* gene expression coincides with decreased host-seeking while high *ILP* expression coincides with increased host-seeking behaviours in both *E.coli* challenged and *P. falciparum* infected mosquitoes. Next, we sought to determine whether a functional connection was evident between *ILP* expression levels and host-seeking. To this end, we treated mosquitoes with anti-*ILP3* or anti-*ILP4* morpholinos, which reduced ILP protein levels *in vitro* by 59% and 89%, respectively. We then measured blood feeding rates in control and anti-*ILP* morpholino-treated groups. Both anti-*ILP3* and anti-*ILP4* morpholino-treated mosquitoes showed a statistically significant reduction in blood feeding propensity compared to controls (Chi-squared test, P < 0.05). When offered an artificial bloodmeal for 15 min, 67.1% of mosquitoes in the control group engorged. However, only 43.2% of mosquitoes in the anti-*ILP3* morpholino-treated group engorged within the same timeframe, representing a greater than 20% reduction relative to controls. Similarly, 56.3% of mosquitoes in the anti-*ILP4* morpholino-treated group took a bloodmeal, a reduction of more than 10% relative to controls.

Effects of *E.coli* challenge on *ILP* expression. Our behavioural assays demonstrated that challenge with heat-killed *E. coli* can lead to dynamic changes in feeding behaviour in a dose- and time-dependent manner. Changes in insulin signalling have been linked to both the immune response and altered food seeking behaviour in *Drosophila* (*14, 15*), providing a potential mechanistic explanation for our previous results. To examine potential long-term

consequences of immune challenge on insulin signalling that coincide with behavioural phenotypes, we measured the expression of *ILP3* and *ILP4* in the mosquito midgut and head on days 6 and 14 post-challenge with *E. coli* (Fig. 2cd). We found that time had a significant effect on the expression of both *ILP3* (F= 7.56, d.f.=1 , P=0.02) and *ILP4* (F=4.67 , d.f.=1 , P=0.05) in the midgut. Specifically, expression of these genes was low during the period of reduced host-seeking response (6 days) and elevated when host-seeking was enhanced (14 days). There was no effect of dose on *ILP* expression and no interaction between dose and time. In the head, we found that time also affected *ILP3* expression (F=6.35 , d.f.=1 , P=0.03), but the pattern observed in the midgut was reversed (Fig. S1). That is, *ILP3* expression was high at 6 days and low at 14 days. No significant changes in *ILP4* expression were observed in the head at any of the time points examined (F=0.1 , d.f.=1 , P>0.05).

The effect of timing on host-seeking patterns. Neither immune challenge (heat-killed *E. coli* only, Wald Chi-Square= 3.48, P=0.06) nor a bloodmeal alone (bloodfed control, Wald Chi-Square=0.47, P=0.49) significantly altered host-seeking propensity compared to unmanipulated controls. As previously reported (*7*), bloodfed females challenged with heat-killed *E. coli* on day 0 exhibited a significantly different phenotype from bloodfed control females (Treatment x Test Period, Wald Chi-Square=10.91, P=0.01). Consistent with the ‘manipulation’ phenotype, females challenged directly after the bloodmeal were less likely to respond to the host on days 6-8 post bloodmeal compared to the response 13-15 days after the bloodmeal. However, this significant change in feeding propensity across the two sample periods was limited to the treatment in which the immune challenge was received on the same day as the bloodmeal (HK-0 between periods, Wald Chi-Square=13.66 , P<0.001). In the other groups, there was no significant difference between feeding propensity in the two time periods tested, nor was there a significant difference between these treatments and the bloodfed controls. There also was no significant difference between the feeding patterns observed for the bloodfed control and the group challenged on day 2 post bloodmeal (Treatment x Stage, Wald Chi-Sqaure=0.17, P=0.68) or the group challenged on day 4 post bloodmeal (Treatment x Stage, Wald Chi-Squared=0.03, P=0.87). See Figure 3a.

Effect of dose on host-seeking patterns. There was no difference between the injury and unmanipulated controls (Dose x Stage, Injury Control/Control Model, Wald Chi-Squared= 0.054, P=0.973) and thus, we combined these treatments for the remainder of analyses. There was a significant effect of replicate on overall response (Wald Chi-Squared=29.82, df=2, P<0.001), but no significant interactions between replicate and other parameters. Controlling for the replicate effect, there was a significant interaction between test period and dose (Wald Chi-squared= 28.14, df=11, P<0.001). See Figure 3b.

In the first test period (6-8 days post-challenge), all challenged mosquitoes were significantly less responsive to host cues than control females (Bonferroni pairwise comparison, P<0.05). The higher the dose of heat-killed *E. coli* a treatment group received, the lower its response (Wald Chi-Squared= 36.44, df=4, P<0.001). Mosquitoes receiving a low dose of heat killed *E. coli* were significantly less likely to respond than mosquitoes receiving no immune challenge (Bonferroni pairwise comparison, P=0.015) and significantly more likely to respond than those receiving the high dose of *E. coli* (Bonferroni pairwise comparison, P=0.032).

When mosquitoes from the same treatment groups were assayed on days 14-16 post bloodmeal, we observed the opposite trend. The response of mosquitoes to host odor increased with the dose of heat-killed *E. coli*. There was a significant difference between the

high and medium dose treatment groups compared to the control (Bonferroni pairwise comparison, P=0.018). There was also a significant difference between the low and high treatment groups, but no significant difference between the medium and the other two treatment groups.

The effect of dose on expression of *DEF1*.We found significant effects of dose, sampling time point, and the interaction between dose and sampling time point (Table S1, Fig S2) on the expression of *DEF1*. Overall, immune challenge relative to no challenge significantly increased the expression of *DEF1* (Adjusted Bonferroni: unmanipulated vs. all other immune challenge groups; p < 0.0001). Further, there was no significant difference between *DEF1* expression resulting from an injury and the *DEF1* expression elicited by intermediate doses of *E. coli*. The expression of *DEF1* peaked at 12 h post injection. There was a significant interaction between dose of immune challenge and sampling time point because the effect of dose on magnitude of the immune response was strongest 12 h post-immune challenge, or the peak of *DEF1* expression (Adjusted Beferonni: 6 hr vs. 12 hr, p = 0.001; 12 hr vs. 24 hr, p = 0.018; 12 hr vs. 48 hr, p < 0.0001).

Figure S1: Analysis of *DEF1* expression in *An. stephensi* challenged with low, medium, and high doses of heat-killed *E. coli*. Immune challenge significantly increased expression of *DEF1*. The effect of dose on expression was most pronounced at peak expression (12 hr).

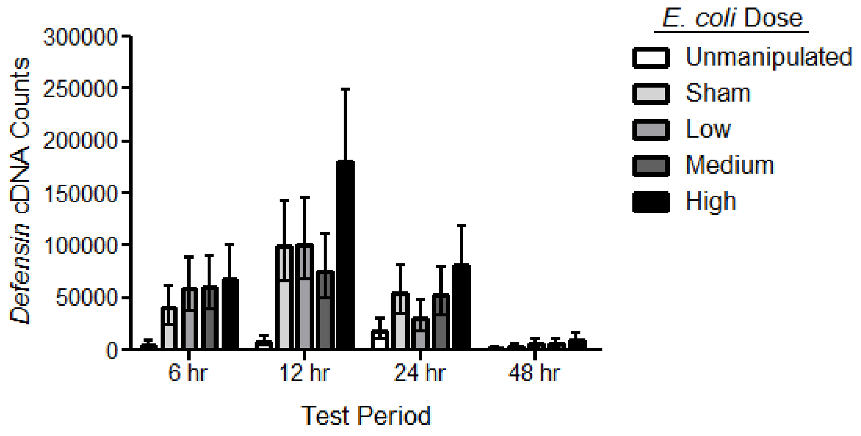

Figure S2: Behavioural sampling with additional intermediate time point. Behavioural responses during an intermediate period (10-12 days post bloodmeal) did not indicate that dose of heat-killed *E. coli* affected the duration or time of decreased response period (dose dependent delay).

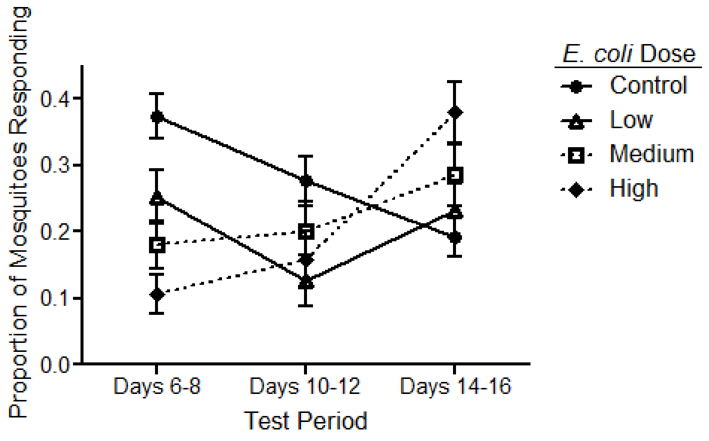

Figure S3: Expression of *ILP3* and *ILP4* in the head tissues of mosquitoes challenged with heat-killed *E. coli* (three doses).Expression of *ILP3* was increased on day 6 in females challenged with heat-killed *E.coli*, while no significant patterns in *ILP4* expression were observed.

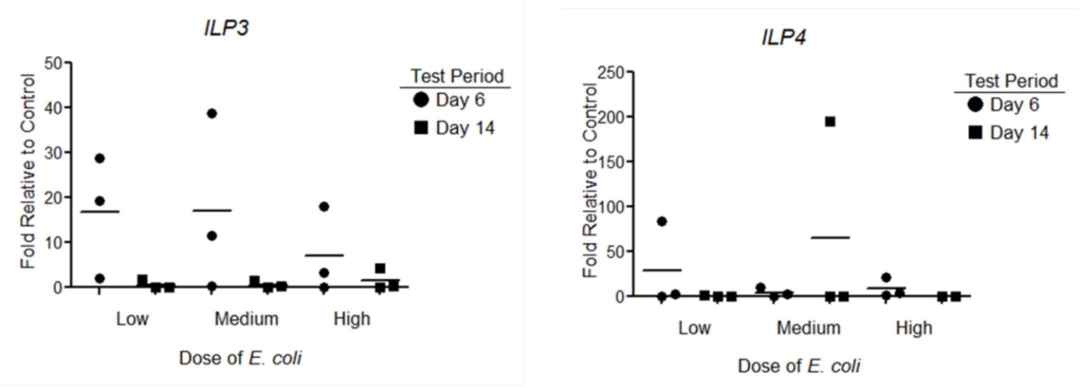

Figure S4. Anti-*AsILP* morpholinos knockdown *As*ILP levels *in vitro* and *in vivo*. Full-length **(A)** *As*ILP3 and **(B)** *As*ILP4 were detected at the highest levels in RIN-5F cells transfected with plasmids for overexpression and treated with control morpholino (Lane 2). Peptides were detected at decreased levels in cells transfected with plasmids for overexpression and treated with anti-As*ILP* morpholinos (Lane 3), but were not detected (ND) in cells transfected with empty plasmid (Lane 1). Values were normalized to Coomassie brilliant blue stain for total protein with proportional levels indicated below the blots.**(C)** *In vivo*, *As*ILP3 and *As*ILP4 were detected at the highest levels in protein extracts from mosquitoes fed control morpholinos and were detected at decreased levels in mosquitoes fed anti-As*ILP* morpholinos. Densitometry values from western blots were normalized to Coomassie brilliant blue stain for total protein and data are represented as mean fold reduction relative to control (broken line, set at 1).

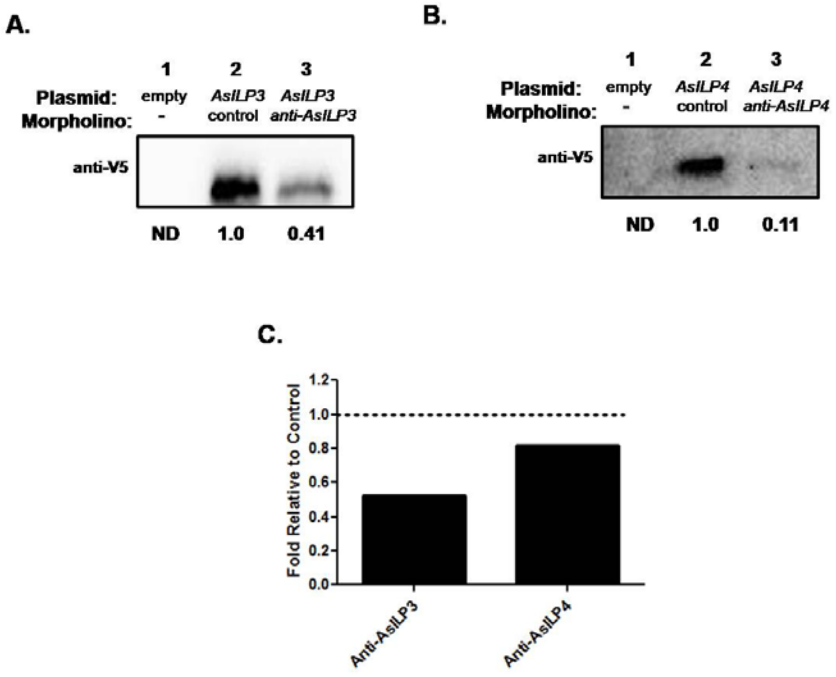

Table S1. Results from mixed effects model analysis demonstrate significant effects of treatment (dose of heat-killed *E. coli*) and sampling time. Replicate was included in the model as a random factor. Variation between samples (mRNA extraction and cDNA conversion) was controlled for using centered *rpS7* cDNA counts.

Factor	F	d.f.	P
Intercept	171.31	1	0.006
treatment	27.80	4	<0.0001
sampling time point	64.85	3	<0.0001
treatment x sampling time point	2.24	12	0.010

Table S2. Prevalence and intensity metrics for infections of *An. stephensi* with *P. falciparum* strain NF54 at UC Davis. Sampling of midguts for expression experiments was destructive. These are replicate infections using the same parasites and mosquito strains used in this work.

Infection	Prevalance	Average Oocyst/Midgut
1	67%	1.83
2	57%	1.75
3	69%	2.00
4	61%	1.41
5	39%	1.36
